# Clinical hypoxemia score for outpatient child pneumonia care lacking pulse oximetry in Africa and South Asia

**DOI:** 10.3389/fped.2023.1233532

**Published:** 2023-10-04

**Authors:** Holly B. Schuh, Shubhada Hooli, Salahuddin Ahmed, Carina King, Arunangshu D. Roy, Norman Lufesi, ASMD Ashraful Islam, Tisungane Mvalo, Nabidul H. Chowdhury, Amy Sarah Ginsburg, Tim Colbourn, William Checkley, Abdullah H. Baqui, Eric D. McCollum

**Affiliations:** ^1^ Global Program in Pediatric Respiratory Sciences, Eudowood Division of Pediatric Respiratory Sciences, Department of Pediatrics, School of Medicine, Johns Hopkins University, Baltimore, MD, United States; ^2^ Department of Epidemiology, Johns Hopkins Bloomberg School of Public Health, Baltimore, MD, United States; ^3^ Division of Emergency Medicine, Department of Pediatrics, Baylor College of Medicine, Houston, TX, United States; ^4^ Projahnmo Research Foundation, Dhaka, Bangladesh; ^5^ Department of Global Public Health, Karolinska Institutet, Stockholm, Sweden; ^6^ Malawi Ministry of Health, Lilongwe, Malawi; ^7^ University of North Carolina (UNC) Project Malawi, Lilongwe, Malawi; ^8^ Department of Pediatrics, UNC, Chapel Hill, NC, United States; ^9^ Clinical Trial Center, University of Washington, Seattle, WA, United States; ^10^ Institute for Global Health, University College London, London, United Kingdom; ^11^ Division of Pulmonary and Critical Care, Department of Medicine, School of Medicine, Johns Hopkins University, Baltimore, MD, United States; ^12^ Center for Global Non-Communicable Disease Research and Training, School of Medicine, Johns Hopkins University, Baltimore, MD, United States; ^13^ Health Systems Program, Department of International Health, Johns Hopkins Bloomberg School of Public Health, Baltimore, MD, United States

**Keywords:** hypoxia, clinical decision rules, pediatrics, primary health care, low-income countries, respiratory tract infection (RTI)

## Abstract

**Background:**

Pulse oximeters are not routinely available in outpatient clinics in low- and middle-income countries. We derived clinical scores to identify hypoxemic child pneumonia.

**Methods:**

This was a retrospective pooled analysis of two outpatient datasets of 3–35 month olds with World Health Organization (WHO)-defined pneumonia in Bangladesh and Malawi. We constructed, internally validated, and compared fit & discrimination of four models predicting SpO_2 _< 93% and <90%: (1) Integrated Management of Childhood Illness guidelines, (2) WHO-composite guidelines, (3) Independent variable least absolute shrinkage and selection operator (LASSO); (4) Composite variable LASSO.

**Results:**

12,712 observations were included. The independent and composite LASSO models discriminated moderately (both C-statistic 0.77) between children with a SpO_2 _< 93% and ≥94%; model predictive capacities remained moderate after adjusting for potential overfitting (C-statistic 0.74 and 0.75). The IMCI and WHO-composite models had poorer discrimination (C-statistic 0.56 and 0.68) and identified 20.6% and 56.8% of SpO_2 _< 93% cases. The highest score stratum of the independent and composite LASSO models identified 46.7% and 49.0% of SpO_2 _< 93% cases. Both LASSO models had similar performance for a SpO_2 _< 90%.

**Conclusions:**

In the absence of pulse oximeters, both LASSO models better identified outpatient hypoxemic pneumonia cases than the WHO guidelines. Score external validation and implementation are needed.

## Introduction

The burden of child pneumonia mortality predominantly occurs in low-income and middle-income countries (LMICs) (1). Hypoxemia—a low blood oxyhemoglobin saturation—conveys increased pneumonia mortality risk, yet most children in LMICs lack pulse oximeter access (2, 3). Pulse oximeters identify hypoxemia by non-invasively measuring the peripheral arterial oxyhemoglobin saturation (SpO_2_) (4). To simplify diagnosis in LMICs the World Health Organization (WHO) Integrated Management of Childhood Illness (IMCI) guidelines consider pneumonia a clinical syndrome (5). Although IMCI recommends oximeter use when available, it also provides guidance for settings without pulse oximetry (5).

There are challenges in the application of the IMCI algorithm for pneumonia management, which have remained largely unchanged since their inception in the mid-1990s. Since then, the guidelines have undergone one technical update that recommends children with *chest indrawing* but without clinical danger signs no longer require hospital referral (6, 7). Evidence suggests that gaps remain with the IMCI case management strategy, especially regarding use of the algorithm without pulse oximetry. Specifically, research reported IMCI missed ∼70% of outpatient child pneumonia cases with a SpO_2 _< 90% in Malawi and ∼90% in Bangladesh (8, 9). However, a stronger emphasis on the integration of pulse oximetry into IMCI guidance—while an important next step—is unlikely to immediately solve this issue as healthcare providers in most high pneumonia burden LMICs lack access to pulse oximeter devices in outpatient settings where children usually present to care first. High quality, affordable devices designed for the needs of infants and children in LMICs are not yet available. As a result, healthcare providers instead rely on clinical signs included in the IMCI guidelines to aid in the management of pneumonia cases. As most children first access health systems at outpatient clinics, improving outpatient hypoxemia identification may be key to reducing LMIC pneumonia mortality (4).

Prior research attempted to determine whether clinical signs accurately identify a SpO_2 _< 90% in hospitalized children as current guidelines limit severe case definitions to SpO_2_ measurements <90% (10, 11). While these hospital-based studies report high specificity of clinical signs for a SpO_2_ < 90%, sensitivity was low (10, 11). Similar studies in outpatient contexts are lacking, and may reveal new evidence than research from hospitals relying on study populations of more severely ill children not representative of outpatient settings. In settings where pulse oximeters are available, recent data also showed elevated mortality among children with a SpO_2_ 90%–92% or 93%, as compared to higher SpO_2_ levels (8, 12–14). These findings further challenge the current guidelines, which recommend SpO_2 _< 90% threshold for hospitalization and oxygen treatment in LMICs.

Given the need for healthcare providers in low-resource settings to rely mainly on IMCI-derived clinical signs for outpatient child pneumonia management, we sought to utilize two unique, contemporary outpatient pediatric pneumonia datasets from Bangladesh and Malawi to accomplish three objectives (15, 16). We first sought to evaluate the performance of IMCI clinical signs for identifying hypoxemia at a higher SpO_2_ threshold (<93%) than recommended. Second, we examined whether other combinations of clinical features not included in IMCI guidelines better identify children with a SpO_2 _< 93%, followed by development and internal validation of pragmatic hypoxemia clinical scores that could be implemented where pulse oximeters remain unavailable. Third, we repeated these analyses using the currently recommended SpO_2 _< 90% threshold.

## Materials and methods

### Settings

We used data from Malawi and Bangladesh. Malawi is an African country with an under 5 mortality rate of 49/1,000 births (17). This study included 18 clinics in Mchinji and Lilongwe districts with a 1.2 million population catchment area, at 1,000–1,100 m altitude (16). From October 2011 to June 2014, non-physician clinicians and nurses at clinics were IMCI trained and documented care of 0–59 month olds with WHO-defined pneumonia (16). Providers used Acare® pulse oximeters with adult clip probes on the big toe if <2 years old or weighing <10 kg (9). Training, data collection, and supervision methodology has been published (16).

Bangladesh has an under 5 mortality rate of 27.3/1,000 live births (18). Since 2001, Projahnmo, a partnership of Johns Hopkins University with the Government of Bangladesh's Ministry of Health and Family Welfare, International Centre for Diarrhoeal Disease Research, Bangladesh, Shimantik, and the Child Health Research Foundation conducted community-based surveillance in Zakiganj subdistrict of Sylhet district in Bangladesh (15). From May 2015 to September 2017 Projahnmo expanded surveillance into two additional subdistricts (Kanaighat, Beanibazar) and augmented IMCI clinic care of three government Upazila Health Complexes (15). Altogether these subdistricts have a 770,000 population at 17–23 m altitude. Upazila Health Complexes provide outpatient and emergency care and limited inpatient pediatric services. IMCI clinic care was provided by Projahnmo physicians per IMCI guidelines (15). From October 2017 Projahnmo physicians measured the SpO_2_ of 3–35 month olds with suspected pneumonia using a Masimo Rad-5® pulse oximeter with a LNCS® Y-I wrap sensor on the big toe. Parent study methodology is published (15).

### Inclusion and exclusion criteria

We generated an analytic sample of healthcare visits from Bangladesh and Malawi datasets. Inclusion criteria were: valid SpO_2_, IMCI-defined non-severe or severe pneumonia (2014 guidelines), (5) and age 3–35 months, as the Bangladesh study population with a SpO_2_ was limited to this age (19). See Supplementary Material for study definitions. Implicit to this analysis we assumed SpO_2_ was unavailable and excluded it from pneumonia definitions.

### Variables

Our primary outcome was a SpO_2 _< 93%; SpO_2 _< 90% was secondary. We explored associations between clinical variables and SpO_2_ ranges to evaluate <93% as the primary outcome. We selected variables *a priori*: sex, age, weight-for-age *z*-score (WAZ), chest indrawing, wheezing, severe respiratory distress (grunting, head nodding, nasal flaring, and/or severe fast breathing), cyanosis, fever (temperature ≥38 °C), and WHO-defined general danger signs (stridor, inability to feed/drink, convulsions, and/or lethargy) (8, 18). Severe fast breathing was defined as follows: respiratory rate ≥60 breaths/min for 3–11 month olds, ≥50 breaths/min for 12–59 month olds (20).

### Analysis

We evaluated missingness using a 5% threshold. We used Chi-squared and Fisher's exact tests for proportions, Wilcoxon rank-sum for non-parametric data, and Student's t-test for normally distributed data comparisons. We reviewed individual-level variables and their associated SpO_2 _< 93% and <90% predictive quality. For SpO_2 _< 93% and <90% model development we randomly split the dataset into derivation (70%) and validation (30%) sets balanced by outcome and country. For each model we fit a logistic regression model with hypoxemia as the binary outcome measure. We allowed selection of *country* as a fixed effect to account for significant differences by country. Thereafter, we used a random intercept in each post selection model to control for country after interrogating the suitability of this approach with the Hausman test (21). All analyses were by Stata 16.1 (StataCorp, College Station, TX).

#### Model development

##### IMCI guidelines (IMCI model) and composite WHO guidelines (WHO-composite model)

The IMCI model reflects 2014 IMCI referral criteria (Supplementary Material) (5). The WHO-composite model is a composite of four WHO guidelines (5, 22–24). We fit both models using the *logistic* command for implementing multivariable, maximum-likelihood logit models to obtain odds ratios (and 95% confidence intervals) comparing the odds of hypoxemia vs. non-hypoxemia.

##### Independent variable LASSO model (independent LASSO model) and composite variable LASSO model (composite LASSO model)

We used the least absolute shrinkage and selection operator (LASSO) reduction method, testing two selection modes [(1) 10-fold cross-validation selection and (2) adaptive selection] using the derivation dataset to develop both models from an expanded variable list of the IMCI and WHO-composite models (Supplementary Material) (25, 26). For Independent LASSO we used *singular* variables (i.e., *independent*) whereas for Composite LASSO we used *composite* variables for “danger signs” (WHO-defined general danger signs) and “severe respiratory distress.” For both models we compared the two methods using the C-statistic based on predicted estimates, sensitivity, and specificity. If we found no statistical difference between methods, we used the selection results from the simplest model to implement an unsupervised approach (i.e., selection of the full variable rather than one category of a three-category variable) to refit both models.

#### Hypoxemia score development and validation

We compared discriminatory power and model fit [C-statistic and Bayesian Information Criteria (BIC)] of the four maximum-likelihood logit models using the derivation dataset models (27, 28). Using an unsupervised approach (i.e., if only two age groups were selected, we retained all age categories), each of the LASSO model covariates were kept on the log scale, rounded to the nearest 0.5, and doubled to form an integer (14, 29, 30). We then split the score into approximately equally sized quintiles to create hypoxemia risk categories.

Using the validation dataset, LASSO model scores were estimated by child, and score discriminatory power to identify children with and without hypoxemia was determined. Scores were not developed using IMCI and WHO-composite models. The C-statistic, sensitivity, specificity, positive and negative predictive value (PPV and NPV), and positive and negative likelihood ratios (LR+ and LR−) were compared across all models. We adjusted C-statistics for optimism by bootstrapping (200 repetitions) to account for any overfitting (31). A C-statistic 0.71–0.80 was considered moderate and >0.80 as excellent discriminatory power (32). We applied the same analysis methodology for SpO_2 _< 90%.

## Results

### Study population

We included 12,712 pneumonia cases; 63.6% (*n* = 8,081) were from Bangladesh (Supplementary Material). Table 1 shows all participant characteristics by SpO_2_. A SpO_2 _< 93% was in 10.4% (1,328/12,712) of cases and 63.6% (845/1,328) of SpO_2 _< 93% cases were in Malawi. Most SpO_2 _< 93% cases had non-severe pneumonia (1,065/1,328; 81.4%) and, without a SpO_2_ measurement, were hospitalization ineligible per IMCI guidelines. A larger proportion of severe (263/1,198, 21.9%) than non-severe (1,065/11,514, 9.2%) cases had a SpO_2 _< 93%. A SpO_2 _< 90% was in 4.6% (602/12,712) of cases and in 3.8% (434/11,514) with non-severe disease. 2014 IMCI hospital referral criteria missed 72.0% (434/602) of SpO_2 _< 90% cases. While Bangladesh and Malawi case characteristics differed in frequency, other than WAZ_ _<_ _−3 and danger signs, they had similar crude associations with a SpO_2 _< 93% (Supplementary Material).

**Table 1:** Patient characteristics by SpO_2 _< 93% ( full dataset, *N* = 12,712).

Characteristics	Total	SpO_2_ ≥ 93%	SpO_2 _< 93%	*p*-value
	*N* = 12,712	*N* = 11,384 (89.6%)	*N* = 1,328 (10.4%)	
Study country, *n* (%)				<0.001
Bangladesh	8,081 (63.6%)	7,598 (66.7%)	483 (36.4%)	
Malawi	4,631 (36.4%)	3,786 (33.3%)	845 (63.6%)	
Child age (months), median (IQR)	12 (6, 19)	12 (6, 20)	10 (6, 17)	<0.001
Child sex, *n* (%)				0.36
Male	6,895 (54.2%)	6,207 (54.5%)	688 (51.8%)	
Female	5,530 (43.5%)	4,950 (43.5%)	580 (43.7%)	
Missing	287 (2.3%)	227 (2.0%)	60 (4.5%)	
Weight for age *z*-score, *n* (%)				0.056
≥ −2.0	9,441 (74.3%)	8,463 (74.3%)	978 (73.6%)	
−3.0≤ *z*-score ≤ −2.0	2,080 (16.4%)	1,899 (16.7%)	181 (13.6%)	
< −3.0	852 (6.7%)	760 (6.7%)	92 (6.9%)	
Missing	339 (2.7%)	262 (2.3%)	77 (5.8%)	
WHO danger signs, *n* (%)	364 (2.9%)	183 (1.6%)	181 (13.6%)	<0.001
Stridor at rest	126 (1.0%)	65 (0.6%)	61 (4.7%)	<0.001
Unable to feed	187 (1.5%)	74 (0.7%)	113 (8.5%)	<0.001
Lethargy or unconscious	58 (0.5%)	29 (0.3%)	29 (2.2%)	<0.001
Convulsions	62 (0.5%)	35 (0.3%)	27 (2.0%)	<0.001
Severe respiratory distress, *n* (%)	2,352 (18.5%)	1,667 (14.6%)	685 (51.6%)	<0.001
Grunting	399 (3.1%)	179 (1.6%)	220 (16.7%)	<0.001
Head nodding	636 (5.0%)	402 (3.5%)	234 (17.8%)	<0.001
Nasal flaring	1,219 (9.6%)	776 (6.8%)	443 (33.5%)	<0.001
Severe fast breathing^a^	993 (7.8%)	739 (6.5%)	254 (19.4%)	<0.001
Central cyanosis, *n* (%)	104 (0.8%)	26 (0.2%)	78 (6.0%)	<0.001
Temperature ≥38° C, *n* (%)	3,540 (28.4%)	3,009 (26.8%)	531 (41.9%)	<0.001
Chest indrawing, *n* (%)	3,944 (31.0%)	3,126 (27.5%)	818 (61.6%)	<0.001
Wheezing, *n* (%)	462 (3.6%)	248 (2.2%)	214 (16.1%)	<0.001
Non-severe IMCI pneumonia^b^, *n* (%)	11,514 (90.8%)	10,449 (91.9%)	1,065 (81.4%)	<0.001
Severe IMCI pneumonia^c^, *n* (%)	1,198 (9.4%)	935 (8.2%)	263 (19.8%)	<0.001

SpO_2_ indicates peripheral arterial oxyhemoglobin saturation; IQR, interquartile range; WHO, World Health Organization; IMCI, integrated management of childhood illnesses.

^a^ Respiratory rate ≥70 breaths/min for 3–11 month olds, ≥60 breaths/min for 12–59 month olds.

^b^ Cough and/or difficult breathing plus fast breathing for age (respiratory rate ≥50 breaths/min for 3–11 month olds, ≥40 breaths/min for 12–59 month olds) or chest wall indrawing without any WHO danger signs (stridor at rest, unable to feed, lethargy or unconscious, convulsions).

^c^ Cough and/or difficult breathing plus at least one WHO danger sign (stridor at rest, unable to feed, lethargy or unconscious, convulsions), WAZ < −3, or HIV-infection with chest indrawing.

We assessed the relationship between referral criteria and hypoxemia at SpO_2 _< 90%, 90%–92%, and <93% for the IMCI and WHO-composite models (Table 2). WAZ_ _≤_ _−3, in both models, was associated with a SpO_2_ 90%–92% and <93% but not <90%. In the WHO-composite model severe respiratory distress was associated with an increased adjusted odds of an abnormal SpO_2_ regardless of SpO_2_ range. While in the IMCI model danger signs were associated with hypoxemia, including respiratory distress in the WHO-composite model attenuated its effect at each SpO_2_ threshold. Respiratory distress was associated with each SpO_2_ threshold in the WHO-composite model.

**Table 2:** Association of IMCI guideline and WHO-composite models with SpO_2_ ranges (full dataset, *N* = 12,712).

Characteristics	SpO_2_ < 90%^a^			SpO_2_ 90%–92%^a^			SpO_2_ < 93%^a^		
	*n* = 11,986			*n* = 12,110			*n* = 12,712		
	aOR	95% CI	*p*-value	aOR	95% CI	*p*-value	aOR	95% CI	*p*-value
IMCI guideline model									
Intercept	0.04	(0.02, 0.10)	<0.001	0.06	(0.03, 0.12)	<0.001	0.11	(0.05, 0.23)	<0.001
WHO danger signs^a^	9.01	(7.08–11.47)	<0.001	2.29	(1.60–3.28)	<0.001	5.70	(4.57–7.12)	<0.001
Weight for age *z*-score ≤ −3	1.24	(0.86–1.80)	0.206	1.51	(1.14–2.01)	0.005	1.43	(1.13–1.81)	0.003
WHO-composite model									
Intercept	0.02	(0.01, 0.05)	<0.001	0.05	(0.03, 0.08)	<0.001	0.07	(0.04, 0.13)	<0.001
WHO danger signs^a^	4.82	(3.72–6.24)	<0.001	1.41	(0.97–2.04)	0.080	3.22	(2.54–4.09)	<0.001
Weight for age z-score ≤ −3	1.20	(0.82–1.74)	0.354	1.47	(1.10–1.97)	0.008	1.40	(1.10–1.78)	0.007
Severe respiratory distress^b^	5.74	(4.77–6.91)	<0.001	3.52	(2.99–4.14)	<0.001	4.59	(4.04–5.20)	<0.001

SpO_2_ indicates peripheral arterial oxyhemoglobin saturation; IMCI, integrated management of childhood illnesses; WHO, World Health Organization.

^a^ Stridor at rest, unable to feed or drink, convulsions, lethargy or unconscious.

^b^ Grunting, nasal flaring, head nodding, or severe fast breathing (≥70 breaths/min among 3–11 month olds, and ≥60 breaths/min among 12–35 month olds).

### Multivariable predictive model comparison and score development

Derivation (*n* = 3,813) and validation (*n* = 8,899) datasets have similar patient characteristic distributions (Supplementary Material, all *p* > 0.05). Table 3 presents adjusted odds ratios (aORs) for SpO_2 _< 93% using model-specific predictors from the derivation dataset. In the independent LASSO model, individual predictor scores ranged from −1 to 3. The composite LASSO model scores ranged from −1 to 4. Overall predictive performance of the independent (C-statistic = 0.774) and composite LASSO models (C-statistic = 0.773) was moderate (Table 3) and did not differ in discriminatory power (*p* = 0.480), with total score ranges in the Supplementary Material. Model fit improved from the IMCI (BIC 5,536.5) and WHO-composite models (BIC 5,155.9) to the independent (BIC 4,650.6) and composite LASSO models (BIC 4,712.2). Overall, the independent (C-statistic = 0.774) and composite LASSO models (C-statistic = 0.773) better discriminated between children with and without a SpO_2 _< 93% than the IMCI (C-statistic = 0.661) and WHO-composite models (C-statistic = 0.734). SpO_2 _< 90% score development and cross-model comparison are in the Supplementary Material.

**Table 3:** Clinical hypoxemia score for identifying a SpO_2 _< 93% (derivation dataset).

Characteristics	IMCI guideline model		WHO-composite model		Independent LASSO model			Composite LASSO model		
	Log (odds)	95% CI	Log (odds)	95% CI	Log (odds)	95% CI	Score	Log (odds)	95% CI	Score
WHO danger signs^a^	1.724**	(1.458, 1.990)	1.140**	(0.853, 1.426)				0.521**	(0.173, 0.870)	1
Weight for age *z*-score ≤ −3	0.321*	(0.039, 0.603)	0.298*	(0.007, 0.588)	0.312*	(0.005, 0.620)	1	0.302^+^	(0, 0.605)	1
Severe respiratory distress^b^			1.564**	(1.413, 1.715)				0.990**	(0.813, 1.168)	2
Grunt	–	–	–	–	0.740**	(0.407, 1.073)	1			–
Nasal flaring	–	–	–	–	0.531**	(0.314, 0.749)	1			–
Head nodding	–	–	–	–	0.788**	(0.523, 1.053)	2			–
Severe fast breathing^c^	–	–	–	–	0.773**	(0.541, 1.004)	2			–
Age categories in months										
3–5	–	–	–	–	Ref		–	Ref		–
6–11	–	–	–	–	−0.057	(0.269, 0.154)	0	−0.069	(−0.278, 0.140)	0
12–23	–	–	–	–	−0.444**	(−0.663, −0.224)	−1	−0.407**	(−0.621, −0.193)	−1
24–35	–	–	–	–	−0.562**	(−0.844, (−0.280)	−1	−0.509**	(−0.785, −0.234)	−1
Unable to feed	–	–	–	–	0.444	(−0.113, 1.001)	1	–	–	–
Lethargy or unconscious	–	–	–	–	−0.332	(1.275, 0.611)	−1	–	–	–
Cyanosis	–	–	–	–	1.701**	(1.052, 2.350)	3	1.763**	(1.171, 2.355)	4
Temperature ≥38° C	–	–	–	–	0.217*	(0.051, 0.383)	0	0.212*	(0.048, 0.375)	0
Chest indrawing	–	–	–	–	1.060**	(0.880, 1.240)	2	1.090**	(0.915, 1.264)	2
Wheezing	–	–	–	–	1.156**	(0.860, 1.453)	2	1.103**	(0.822, 1.3840	2
B_0_ intercept	−2.246**	(−3.015, −1.476)	−2.692**	(−3.328, −2.057)	−2.855**	(−3.597, −2.113)	–	−2.905**	(−3.635, −2.175)	–
*N*	8,899		8,899		8,675			8,724		
C-statistic	0.560	(0.546, 0.573)	0.702	(0.684, 0.719)	0.774	0.756, 0.792		0.773	0.755, 0.791	
BIC	5,536.5		5,155.9		4,650.6			4,712.2		

SpO_2_ indicates peripheral arterial oxyhemoglobin saturation; IMCI, integrated management of childhood illnesses; WHO, World Health Organization; LASSO, least absolute shrinkage and selection operator reduction method for selection; all models represented here were fit using mixed effects logistic regression with a random effect for country (after selection and refitting for independent and composite LASSO models); BIC, Bayesian information criterion.

All models include a random effect to account for the clustering effect of country.

+ *p* < 0.1.

* *p* < 0.05.

** *p* < 0.001.

^a^ Stridor at rest, unable to feed or drink, convulsions, or lethargy or unconsciousness.

^b^ Grunting, nasal flaring, head nodding, or severe fast breathing.

^c^ Respiratory rate of ≥70 breaths/min if 3–11 months old; ≥60 breaths/min if 12–35 months old.

### Clinical score performance—validation dataset

**Figure 1:**
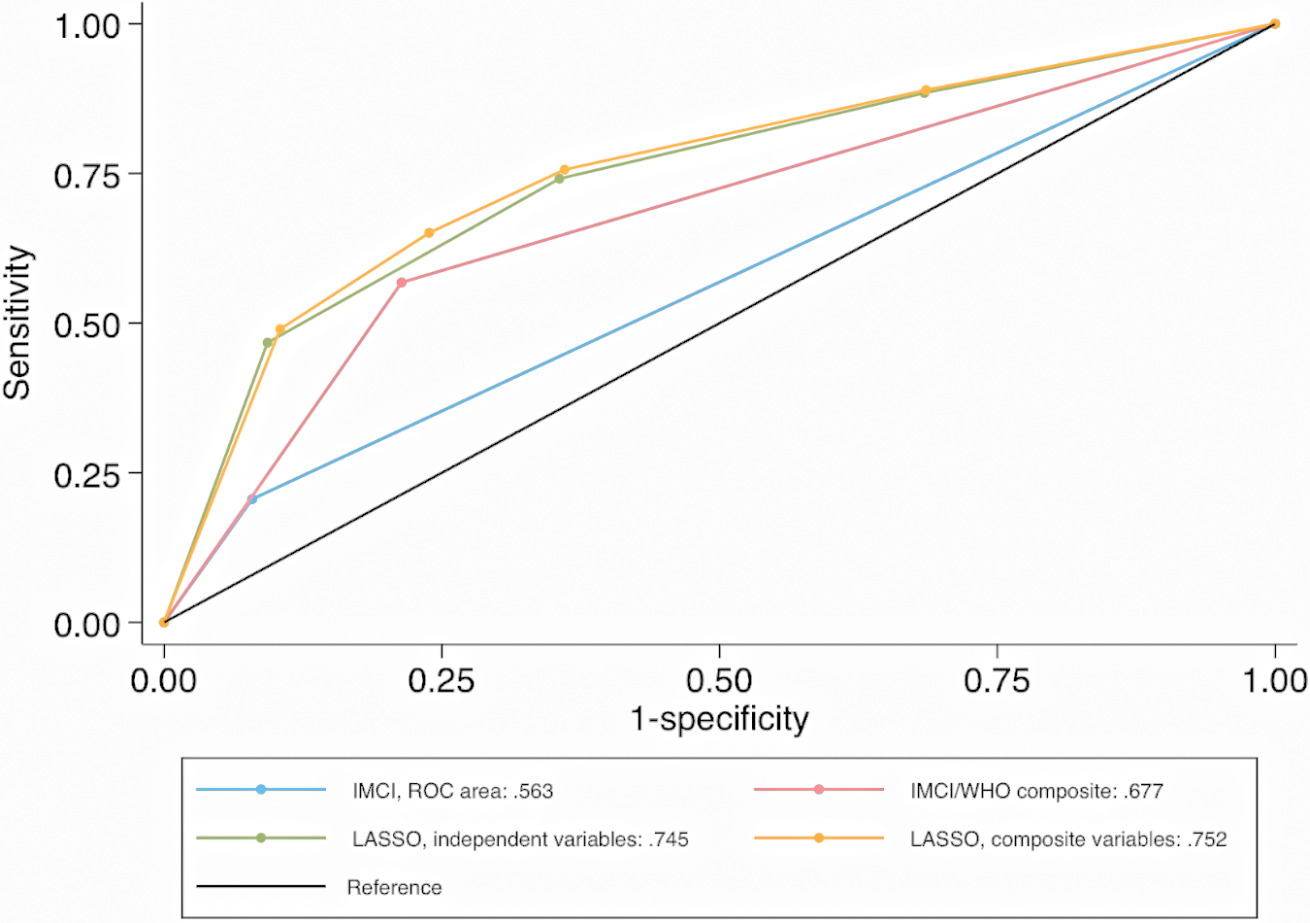
Comparison of ROC curves for identifying SpO_2 _< 93% cases (validation dataset). ROC, receiver operating characteristic curve; SpO_2_, peripheral arterial oxyhemoglobin saturation; IMCI, integrated management of childhood illnesses; WHO, World Health Organization; LASSO, least absolute shrinkage and selection operator reduction method.

In the validation dataset, independent (C-statistic = 0.745) and composite LASSO model (C-statistic = 0.752) scores moderately discriminated between SpO_2 _< 93% and ≥94% cases (Figure 1). When examining the C-statistics adjusted for optimism, there was minimal C-statistic change for both scores (Table 4), and independent and composite LASSO model discriminatory power did not differ (*p* = 0.480). For the independent and composite LASSO models about 4% of children with a score in the first stratum had SpO_2 _< 93% compared to >35% in the last stratum (Table 4).

**Table 4:** Model performance for identifying a SpO_2 _< 93% (validation dataset).

	Hypoxemia risk (*n*/*N*)^a^	Crude OR (95% CI)	Mean predicted hypoxemia, %	C-statistic (adjusted for optimism;^b^ 95% CI)	Sensitivity	Specificity	PPV	NPV
IMCI guideline model								
Non-case	316/3,460	Ref	9.1%	0.563 (0.564; 0.528, 0.600)	20.6%	92.1%	23.2%	90.9%
Case	82/353	3.011 (2.292, 3.955)	23.2%	–				
Total	398/3,813	–		–				
WHO-composite model								
Non-case	172/2,857	Ref	6.0%	0.677 (0.677; 0.635, 0.712)	56.8%	78.6%	23.6%	94.0%
Case	226/956	4.833 (3.900, 5.989)	23.6%	–				
Total	398/3,813	–		–				
Independent LASSO model								
−2 to −1	46/1,124	Ref	4.1%	0.745 (0.742; 0.696, 0.779)	46.7%	90.7%	36.9%	93.6%
0	57/1,179	1.191 (.800, 1.771)	4.8%	–				
1–2	109/1,006	2.848 (1.995, 4.065)	10.8%	–				
3–13	186/504	13.707 (9.697, 19.376)	36.9%	–				
Total	398/3,813			–				
Composite LASSO model								
−1	44/1,117	Ref	3.9%	0.752 (0.750; 0.701, 0.787)	49.0%	89.5%	35.3%	93.8%
0	53/1,164	1.163 (.773, 1.750)	4.6%	–				
1	42/458	2.462 (1.589, 3.814)	9.2%	–				
2	64/522	3.408 (2.286, 5.079)	12.3%	–				
3–11	195/552	13.320 (9.402, 18.871)	35.3%	–				
Total	398/3,813							

SpO_2_ indicates peripheral arterial oxyhemoglobin saturation; OR, odds ratio; CI, confidence interval; PPV, positive predictive value; NPV, negative predictive value; IMCI, integrated management of childhood illnesses; WHO, World Health Organization; LASSO, least absolute shrinkage and selection operator reduction method for selection; all models represented here were fit using mixed effects logistic regression with a random effect for country (after selection and refitting for the independent and composite LASSO models).

Hypoxemia risk: numerator (number of hypoxemia cases) and denominator (total number of children with the relevant clinical score).

The independent and composite LASSO models AUC compared using Delong method with Bonferroni correction (*p* = 0.480).

^a^ *n* is the number of observed hypoxemic participants; *N* is the total number of participants.

^b^ C-statistics are adjusted for optimism.

### Cross-model comparison: discriminating between hypoxemic and non-hypoxemic children

Among IMCI and WHO-composite model classified cases, 23% and 24% had a SpO_2 _< 93%, while among non-cases 9% and 6% had a SpO_2 _< 93% (Table 4). The ability to discriminate between SpO_2 _< 93% and ≥94% cases using the IMCI (C-statistic = 0.563) and WHO-composite model (C-statistic = 0.677) criteria was low. Based on model fit and discrimination, the composite LASSO model was the most predictive of SpO_2 _< 93%, identifying 49.0% of SpO_2 _< 93% cases. The independent LASSO model performed similarly, followed by WHO-composite and IMCI models. SpO_2 _< 90% results on score validation and ability to discriminate hypoxemia from non-hypoxemia cases are in the Supplementary Material.

### Clinical signs for a SpO_2_ < 93%

The Supplementary Material includes the diagnostic performance of individual clinical signs for a SpO_2__ _< 93% and <90% using the validation datasets.

## Discussion

Using pooled data from 12,712 IMCI-defined child pneumonia cases evaluated at 21 clinics in Malawi and Bangladesh we examined WHO IMCI guideline hypoxemia identification performance and developed and internally validated clinical hypoxemia scores for use in LMICs where pulse oximeters suitable for pediatric outpatient care are scarce. Our findings suggest more hypoxemic children could be identified during outpatient care lacking pulse oximeters if additional signs of respiratory distress are incorporated into IMCI. Notably, >80% of hypoxemic cases with a SpO_2 _< 93% and >70% with a SpO_2 _< 90% were misclassified by IMCI as ineligible for hospital referral when pulse oximeters are unavailable. The independent LASSO model added age, severe respiratory distress, chest indrawing, cyanosis, fever, and wheezing parameters into the base IMCI guideline model and better identified children with a SpO_2 _< 93% than the IMCI and WHO-composite models. The composite LASSO model's simplified composite variables may facilitate implementation. Both LASSO models also achieved excellent discrimination of SpO_2 _< 90% cases.

Unlike other studies evaluating hypoxemia predictors we focused on ambulatory rather than hospital settings. This distinction is important as our findings should therefore be generalizable to outpatient settings without oximeters and with lower hypoxemia prevalence than hospitals (33, 34). Notably, individual variable sensitivity and specificity for hypoxemia are largely similar to hospital-based studies (11). However, our WHO-composite model and two LASSO models have good to excellent discriminatory values distinguishing between hypoxemic and non-hypoxemic pneumonia cases, contrasting to other work with smaller samples that limited analyses (11). An exception is a large, multi-center, hospital-based Nigerian study that found a combination of respiratory distress, inability to feed, cyanosis, lethargy and severe tachypnea had lower discrimination (C-statistic = 0.655) for SpO_2 _< 90% amongst respiratory and non-respiratory cases than in our data (10). The lower C-statistic may reflect the authors inclusion of older children <15 years.

It is also important our results are interpreted within the broader child pneumonia context of known mortality risk factors. In the IMCI and WHO-composite models WAZ ≤ −3 was not associated with a SpO_2 _< 90%, yet is a known mortality risk factor (14, 35). Conversely wheezing was retained in both LASSO models but, when identified alone without accompanying respiratory distress, it is not associated with radiographic pneumonia or in-hospital mortality (Supplementary Material) (14, 36). Isolated wheeze usually reflects milder, self-limited viral illness (e.g., bronchiolitis) and is susceptible to non-differential misclassification when confused with transmitted upper respiratory sounds.

While our pooling of data from studies in South Asia and Africa aimed to improve generalizability, there are several differences between the settings and studies that may be limitations. Malawi data had a higher frequency of hypoxemia, danger signs, and respiratory distress than Bangladesh data. Alternatively, WAZ ≤ −3 was more frequent in Bangladesh than Malawi. These differences, in part, may reflect higher HIV and malaria prevalence or modestly higher altitude in Malawi. These diseases increase susceptibility for hypoxemia or, for malaria, increase the frequency of signs overlapping with IMCI pneumonia (37). Although the designs differed, all personnel were similarly trained per IMCI and followed similar data collection procedures. Two different pulse oximeter devices were used in each study. In Malawi, healthcare workers used Acare pulse oximeters with the adult clip probes while in Bangladesh study staff used Masimo Rad5 pulse oximeters with wrap sensors. Although the devices differed, healthcare workers followed similar measurement procedures that relied on SpO_2_ measurement of the child's big toe, which mitigated probe fit issues with the Acare device. Additionally, while pulse oximeter manufacturer algorithms for SpO_2_ calculation may differ, we do not expect systematic, clinically relevant differences in population-level SpO_2_ summary statistics between these devices as both were used similarly on patients by frequently supervised healthcare workers, met ISO 80601-2-61:2017 requirements for the basic safety and essential accuracy performance of pulse oximeters during laboratory testing, are CE marked, and commercially available. Lastly, the under 5 mortality rate in Malawi is higher than Bangladesh, (17, 18) which aligns with our results suggesting Malawi cases were more severe. Nevertheless, we attempted to account for unmeasured confounders from epidemiological, health system, and methodological differences by fitting models with a country variable.

We recognize that the future use of a clinical hypoxemia score would require further external validation and evaluation of implementation acceptability, feasibility, fidelity, and other early implementation indicators. Broader use of such clinical scores largely depend on health system capacity—for example, developing and maintaining well-trained clinical staff who can recognize the clinical features that comprise the score and who accept new guidelines, local expertise for overseeing and advising any adapted implementation, and, most importantly, resources such as oxygen and antibiotics to act on these symptoms and improve care with resultant improved outcomes. To optimize implementation further studies are needed to examine and adapt to changes to clinical procedure flows as well as assessment and adaptation of health system dynamics that include the capacity to handle additional referrals. These steps should be conducted within the context of strengthening the outpatient system for subsequent pulse oximeter implementation when appropriate, high quality devices for infants and children become accessible (38).

In sum, these findings may improve hypoxemic pneumonia identification in clinics either without pulse oximetry at all or lacking pulse oximeters suitable for pediatric use. Given a SpO_2_ < 93% is highly associated with mortality, (3, 12, 14) earlier hypoxemic case identification with successful referral may reduce fatality. While these models could advance care for hypoxemic children they are an inadequate substitution for pulse oximeters. Pulse oximetry scale up in ambulatory settings must be prioritized, as should pulse oximeter device development targeted for young children in LMICs. Where pulse oximetry is unavailable for children, our models suggest children with chest indrawing and/or with other signs of respiratory distress could be referred. To successfully implement these models ambulatory health workers of varying pediatric experience will need further training to recognize signs of respiratory distress in children. Understanding how these changes may affect referral quality, uptake, and hospitalizations is critical. Next steps include external validation and research evaluating implementation feasibility of the hypoxemia scores.

## Data Availability

The datasets presented in this article are not readily available because of country limits on data availability. Requested to access the datasets should be directed to EM, emccoll3@jhmi.edu.
