# Supplementary material for: Clinical hypoxemia score for outpatient child pneumonia care lacking pulse oximetry in Africa and South Asia
Source: Front Pediatr. 2023 Oct 4;11:1233532. doi: 10.3389/fped.2023.1233532 (PMC10582699; doi:10.3389/fped.2023.1233532)
Supplement: Supplementary file 1 [file Datasheet1.pdf]

Supplemental Material

Title: Clinical hypoxemia score for outpatient child pneumonia care lacking pulse oximetry in Africa and South Asia

Corresponding Author: Dr. Eric D. McCollum, MD, MPH; email: emccoll3@jhmi.edu

Supplemental Material Contents:

Supplemental Figure 1: Study Definitions – page 3 [tiff separately attached]

Supplemental Figure 2: Study flowchart – page 4 [tiff separately attached]

Supplemental Table 1: Bangladesh: Risk factors for a SpO_2<93% – page 5

Supplemental Table 2. Malawi: Risk factors for a SpO_2<93% – page 6

Supplemental Table 3. Patient characteristics by SpO_2<93%, stratified by development versus validation dataset – page 7

Supplemental Table 4. Independent LASSO model scores and associated hypoxemia risk (SpO_2<93%), sensitivity, specificity, and positive/negative likelihood ratios (validation dataset) – page 8

Supplemental Table 5. Composite LASSO model scores and associated hypoxemia risk (SpO_2<93%), sensitivity, specificity, and positive/negative likelihood ratios (validation dataset) – page 8

Supplemental Figure 3. Comparison of ROC curves for identifying SpO_2<90% cases (validation dataset) – page 9 [tiff separately attached]

Supplemental Table 6. Association of Models with SpO_2<90% and performance for identifying SpO_2 <90% (development dataset) – page 10

Supplemental Table 7. Independent LASSO model scores and associated hypoxemia risk ($SpO_2<90\%$), sensitivity, specificity, and positive/negative likelihood ratios (validation dataset) – page 11

Supplemental Table 8. Composite LASSO model scores and associated hypoxemia risk ($SpO_2<90\%$), sensitivity, specificity, and positive/negative likelihood ratios (validation dataset) – page 11

Supplemental Table 9. Model performance and hypoxemia ($SpO_2<90\%$) case rate (validation dataset) – page 12

Supplemental Table 10. Performance of clinical signs for a $SpO_2<93\%$ during outpatient pediatric care in Bangladesh and Malawi (validation dataset) – page 13

Supplemental Table 11. Performance of clinical signs for a $SpO_2<90\%$ during outpatient pediatric care in Bangladesh and Malawi (validation dataset) – page 16

Supplemental Table 12. Characteristics of hypoxemic ($SpO_2<93\%$) children indicated as wheezing (n=214)

Supplemental Figure 1. Study Definitions
[separately attached]

*LASSO selection (Independent and Composite LASSO models): starting list included WHO danger signs (independent or composite), severe respiratory distress (independent or composite), central cyanosis, fever, chest indrawing, and wheezing. The reduction method selected the variables listed in Supplemental Figure 1. Reference to “WHO danger signs” or “severe respiratory distress” means use of the composite term/variable.

Supplemental Figure 2. Study flowchart
[Separately attached]

Supplemental Table 1. Bangladesh: Risk factors for a SpO_2 <93% (full dataset, N=8,081)

	$SpO_2 \geq 93\%$ N=7,598	SpO_2 <93% N=483	Crude odds Ratio	95% CI	p-value
Age (months)					
3-5	1,617 (91.8%)	144 (8.2%)	Ref		
6-11	2,249 (93.2%)	164 (6.8%)	0.82*	(0.65 - 1.03)	0.092
12-23	2,500 (95.2%)	127 (4.8%)	0.57***	(0.45 - 0.73)	<0.001
24-35	1,232 (96.2%)	48 (3.8%)	0.44***	(0.31 - 0.61)	<0.001
Child sex					
Male	4,414 (94.4%)	263 (5.6%)	Ref		
Female	3,184 (93.5%)	220 (6.5%)	1.16	(0.96 - 1.40)	0.116
Weight for age z-score (WAZ)					
WAZ ≥ -2.0	5,387 (94.4%)	322 (5.6%)	Ref		
-3.0 < WAZ < -2.0	1,561 (94.1%)	97 (5.9%)	1.04	(0.82 - 1.31)	0.745
WAZ ≤ -3.0	650 (91.0%)	64 (9.0%)	1.65***	(1.24 - 2.18)	<0.001
Fever	1,907 (25.1%)	196 (40.6%)	2.04***	(1.69 - 2.46)	<0.001
WHO danger signs	21 (0.3%)	3 (0.6%)	2.26	(0.67 - 7.59)	0.189
Stridor	0 (0.0%)	0 (0.0%)	-		-
Unable to feed	0 (0.0%)	0 (0.0%)	-		-
Convulsions	3 (0.0%)	1 (0.2%)	2.63	(0.77 - 8.97)	0.122
Lethargy or unconscious	18 (0.2%)	3 (0.6%)	5.25	(0.55 - 50.59)	0.151
Severe respiratory distress	851 (11.2%)	186 (38.5%)	5.24***	(4.26 - 6.44)	<0.001
Grunting	26 (0.3%)	13 (2.7%)	8.06***	(4.11 - 15.78)	<0.001
Nasal flaring	367 (4.8%)	92 (19.0%)	4.64***	(3.61 - 5.95)	<0.001
Head nodding	317 (4.2%)	96 (19.9%)	5.70***	(4.44 - 7.32)	<0.001
Severe fast breathing[a]	344 (4.5%)	79 (16.4%)	4.12***	(3.17 - 5.37)	<0.001
Central cyanosis	7 (0.1%)	7 (1.4%)	15.95***	(5.57 - 45.65)	<0.001
Chest indrawing	2,493 (32.8%)	307 (63.6%)	3.57***	(2.95 - 4.33)	<0.001
Wheezing	25 (0.3%)	12 (2.5%)	7.72***	(3.85 - 15.46)	<0.001

*** p<0.001, ** p<0.05, * p<0.1; Severe fast breathing defined as: ≥70 breaths/min among 3-11 month olds, and ≥60 breaths/min among 12-35 month olds; Fever defined as body temperature ≥38 degrees Celsius

Supplemental Table 2. Malawi: Risk factors for a SpO_2 <93% (full dataset, N=4,631)

	**Not hypoxemic** N=3,786	**Hypoxemic** N=845	**Crude odds Ratio**	**95% CI**	**p-value**
Age (months)					
3-5	617 (79.3%)	161 (20.7%)	Ref		
6-11	1,108 (79.7%)	283 (20.3%)	0.98	(0.79 - 1.22)	0.847
12-23	1,404 (82.7%)	294 (17.3%)	0.80**	(0.65 - 0.99)	0.044
24-35	657 (86.0%)	107 (14.0%)	0.62***	(0.48 - 0.82)	0.001
Child sex					
Male	1,793 (80.8%)	425 (19.2%)	Ref		
Female	1,766 (83.1%)	360 (16.9%)	0.86*	(0.74 - 1.00)	0.057
Missing	227 (79.1%)	60 (20.9%)			
Weight for age z-score (WAZ)					
WAZ ≥ -2.0	3,076 (82.4%)	656 (17.6%)	Ref		
-3.0 < WAZ < -2.0	338 (80.1%)	84 (19.9%)	1.17	(0.90 - 1.50)	0.237
WAZ ≤ -3.0	110 (79.7%)	28 (20.3%)	1.19	(0.78 - 1.82)	0.413
Missing	262 (77.3%)	77 (22.7%)			
Fever, temp>=38c	1,102 (30.5%)	335 (42.8%)	1.71***	(1.46 - 2.00)	<0.0001
WHO danger signs	162 (4.3%)	178 (21.1%)	5.97***	(4.75 - 7.50)	<0.0001
Stridor	65 (1.7%)	61 (7.5%)	4.56***	(3.19 - 6.53)	<0.0001
Unable to feed	74 (2.0%)	113 (13.5%)	7.78***	(5.75 - 10.55)	<0.0001
Convulsions	17 (0.5%)	24 (2.9%)	6.54***	(3.50 - 12.22)	<0.0001
No movement / lethargy	26 (0.7%)	28 (3.3%)	4.99***	(2.91 - 8.55)	<0.0001
Severe respiratory distress	816 (21.6%)	499 (59.1%)	7.14***	(6.06 - 8.41)	<0.0001
Grunting	153 (4.1%)	207 (24.9%)	7.83***	(6.25 - 9.82)	<0.0001
Nasal flaring	409 (10.8%)	351 (41.9%)	5.93***	(5.00 - 7.04)	<0.0001
Head nodding	85 (2.3%)	138 (16.6%)	8.62***	(6.50 - 11.43)	<0.0001
Severe fast breathing	395 (10.5%)	175 (21.2%)	2.30***	(1.89 - 2.80)	<0.0001
Central cyanosis	19 (0.5%)	71 (8.6%)	18.49***	(11.08 - 30.85)	<0.0001
Chest indrawing	633 (16.7%)	511 (60.5%)	7.64***	(6.49 - 8.98)	<0.0001
Wheezing	223 (5.9%)	202 (23.9%)	5.02***	(4.08 - 6.18)	<0.0001

*** p<0.001, ** p<0.05, * p<0.1; Severe fast breathing defined as: ≥70 breaths/min among 3-11 month olds, and ≥60 breaths/min among 12-35 month olds; Fever defined as body temperature ≥38 degrees Celsius

Supplemental Table 3. Patient characteristics by SpO_2<93%, stratified by development versus validation dataset

	Total	Development	Validation	p-value
	N=12,712	N=8,899	N=3,813	
Study country				1.00
Bangladesh	8,081 (63.6%)	5,657 (63.6%)	2,424 (63.6%)	
Malawi	4,631 (36.4%)	3,242 (36.4%)	1,389 (36.4%)	
Child age (months)	12 (6-19)	11 (6-19)	12 (6-19)	0.20
Child sex				0.41
Male	6,895 (54.2%)	4,802 (54.0%)	2,093 (54.9%)	
Female	5,530 (43.5%)	3,890 (43.7%)	1,640 (43.0%)	
Missing	287 (2.3%)	207 (2.3%)	80 (2.1%)	
Weight for age z-score (WAZ)				0.66
WAZ ≥ -2.0	9,441 (74.3%)	6,585 (74.0%)	2,856 (74.9%)	
-3.0 < WAZ < -2.0	2,080 (16.4%)	1,468 (16.5%)	612 (16.1%)	
WAZ ≤ -3.0	852 (6.7%)	603 (6.8%)	249 (6.5%)	
Missing	339 (2.7%)	243 (2.7%)	96 (2.5%)	
WHO danger signs	364 (2.9%)	252 (2.8%)	112 (2.9%)	0.74
Stridor	126 (1.0%)	84 (0.9%)	42 (1.1%)	0.41
Unable to feed	187 (1.5%)	124 (1.4%)	63 (1.7%)	0.27
Lethargy	58 (0.5%)	41 (0.5%)	17 (0.4%)	0.91
Seizures	62 (0.5%)	46 (0.5%)	16 (0.4%)	0.47
Severe respiratory distress	2,352 (18.5%)	1,630 (18.3%)	722 (18.9%)	0.41
Grunting	399 (3.1%)	273 (3.1%)	126 (3.3%)	0.49
Head nodding	636 (5.0%)	440 (5.0%)	196 (5.2%)	0.65
Nasal flaring	1,219 (9.6%)	855 (9.6%)	364 (9.6%)	0.91
Severe fast breathing	993 (7.8%)	687 (7.7%)	306 (8.1%)	0.55
Central cyanosis	104 (0.8%)	73 (0.8%)	31 (0.8%)	0.96
Fever	3,540 (28.4%)	2,477 (28.3%)	1,063 (28.5%)	0.81
Chest indrawing	3,944 (31.0%)	2,751 (30.9%)	1,193 (31.3%)	0.68
Wheezing	462 (3.6%)	319 (3.6%)	143 (3.8%)	0.65
Non-severe pneumonia	11,514 (90.8%)	8,054 (90.7%)	3,460 (91.1%)	0.55
Severe pneumonia	1,198 (9.4%)	845 (9.5%)	353 (9.3%)	0.67

Severe fast breathing defined as: ≥70 breaths/min among 3-11 month olds, and ≥60 breaths/min among 12-35 month olds; Fever defined as body temperature ≥38 degrees Celsius

Supplemental Table 4. Independent LASSO model scores and associated hypoxemia risk (SpO_2<93%), sensitivity, specificity, and positive/negative likelihood ratios (validation dataset)

Score	Hypoxemia risk	Sensitivity	Specificity	LR+	LR-
-2	0	0	100	0.00	1.00
-1	29	12	68	0.37	1.29
0	31	14	67	0.44	1.28
1	12	11	87	0.85	1.02
2	14	17	86	1.22	0.97
3	4	8	96	2.05	0.96
4	4	17	97	5.78	0.86
5	2	9	99	6.67	0.92
6	1	5	99	9.53	0.95
7	1	4	100	10.73	0.97
8	0	2	100	17.16	0.98
9	0	1	100	17.16	0.99
10	0	1	100	.	0.99
11	0	0	100	8.58	1.00

Supplemental Table 5. Composite LASSO model scores and associated hypoxemia risk (SpO_2<93%), sensitivity, specificity, and positive/negative likelihood ratios (validation dataset)

Score	Hypoxemia risk	Sensitivity	Specificity	LR+	LR-
-1	29	11	69	0.35	1.30
0	31	13	67	0.41	1.28
1	12	11	88	0.87	1.02
2	14	16	87	1.20	0.97
3	5	14	96	3.13	0.90
4	6	16	95	3.37	0.89
5	2	9	99	10.80	0.92
6	1	6	100	12.58	0.95
7	0	2	100	13.73	0.98
8	0	2	100	25.74	0.99
9	0	2	100	60.06	0.98
10	0	1	100	17.16	1.00

Supplemental Figure 3. Comparison of ROC curves for identifying SpO_2 <90% cases (validation dataset)

[Separately attached]

Supplemental Table 6. Association of Models with SpO_2 <90% and performance for identifying SpO_2 <90% (development dataset)

	IMCI model		WHO-composite model		Independent LASSO model			Composite LASSO model		
Predictors	log(odds)	95% CI	log(odds)	95% CI	log(odds)	95% CI	Score	log(odds)	95% CI	Score
WHO danger sign, yes	2.247**	(1.964,2.530)	1.649**	(1.345,1.953)	-	-	-	1.005**	(.626,1.384)	2
WAZ < -3, yes	0.290	(-.140,.720)	0.297	(-0.139,.733)	-	-	-	-	-	-
Severe respiratory distress	-	-	1.711**	(1.490,1.931)	-	-	-	1.054**	(.789,1.319)	2
Grunt	-	-	-	-	0.581**	(.204,.959)	1			-
Nasal flaring	-	-	-	-	0.599**	(.309,.890)	1			-
Head nodding	-	-	-	-	0.949**	(.621,1.278)	2			-
Severe fast breathing	-	-	-	-	0.907**	(.600,1.215)	2			-
Age (months)	-	-	-	-	-	-	-			-
3-5	-	-	-	-	Ref		-	Ref		-
6-11	-	-	-	-	-0.111	(-.420,.198)	0	-1.02	(-.403,.199)	-2
12-23	-	-	-	-	-0.608**	(-.936,-.280)	-1	-0.532**	(-.847,-.217)	-1
24-35	-	-	-	-	-0.738**	(-1.168,-.308)	-1	-0.587**	(-.996,-.177)	-1
Unable to feed	-	-	-	-	0.351	(-0.210,.911)	1	-	-	-
No movement / lethargy	-	-	-	-	-0.332	(-1.275,0.611)	-1	-	-	-
Cyanosis	-	-	-	-	2.503**	(1.874,3.133)	5	2.224**	(1.645,2.802)	4
Fever	-	-	-	-	0.186	(-.060,.432)	0	0.220+	(-.019,.459)	0
Chest indrawing	-	-	-	-	1.459**	(1.171,1.747)	3	1.467**	(1.191,1.743)	3
Wheezing	-	-	-	-	-	-	-	-	-	-
B_0, intercept	-3.272**	(-4.185,-2.360)	-3.838**	(-4.580,-3.097)	-4.110	(-5.081,-3.138)	-	-4.168**	(-5.082,-3.254)	-
N	8899		8899		8669			8713		
C-statistic	0.612	(0.589, 0.635)	0.761	(0.736, 0.786)	0.832	(0.809, 0.855)		0.834	(0.812, 0.856)	
BIC	2995.3		2776.5		2350.1			2423.9		

+ p<0.1, * p<0.05, ** p<0.001 Severe respiratory distress: grunting, nasal flaring, head nodding, or severe fast breathing; WHO danger signs: stridor, unable to feed/drink, convulsions, or lethargy or unconsciousness. Severe fast breathing: breaths/min $\geq$70 3-11months; $\geq$60 12-35months. Fever: $\geq$38. "-" indicates where variable is not specified. LASSO, Least Absolute Shrinkage and Selection Operator reduction method for selection; all models represented here were fit using mixed effects logistic regression with a random effect for country (after selection and refitting for Model 3 and 4).

Supplemental Table 7. Independent LASSO model scores and associated hypoxemia risk (SpO_2<90%), sensitivity, specificity, and positive/negative likelihood ratios (validation dataset)

Score	Hypoxemia risk	Sensitivity	Specificity	LR+	LR-
-2	0	0	100	0.00	1.00
-1	33	8	65	0.24	1.40
0	31	13	69	0.41	1.27
1	4	6	96	1.36	0.98
2	9	8	91	0.86	1.01
3	12	13	88	1.03	1.00
4	4	9	96	2.36	0.95
5	3	11	97	3.88	0.92
6	1	11	99	11.98	0.90
7	1	8	100	17.81	0.92
8	1	6	100	20.18	0.95
9	0	2	100	80.73	0.98
10	0	3	100	50.46	0.97
11	0	1	100	20.18	0.99
12	0	1	100	40.37	0.99
13	0	2	100	.	0.98

Supplemental Table 8. Composite LASSO model scores and associated hypoxemia risk (SpO_2<90%), sensitivity, specificity, and positive/negative likelihood ratios (validation dataset)

Score	Hypoxemia risk	Sensitivity	Specificity	LR+	LR-
-2	19	7	80	0.37	1.15
-1	33	7	65	0.21	1.42
0	12	5	88	0.41	1.08
1	11	13	90	1.22	0.97
2	9	8	91	0.98	1.00
3	7	13	93	1.81	0.94
4	5	17	96	4.12	0.86
5	3	13	97	5.10	0.89
6	1	6	100	12.61	0.95
7	0	4	100	20.18	0.96
8	0	2	100	80.73	0.98
9	0	1	100	20.18	0.99
10	0	2	100	60.55	0.98
11	0	1	100	.	0.99

Supplemental Table 9. Model performance and hypoxemia (SpO_2 <90%) case rate (validation dataset)

	Hypoxemia risk (n/N)	Crude OR (95% CI)	Mean predicted hypoxemia, %	C-statistic (adjusted for Optimism; 95% CI)	Sensitivity	Specificity	PPV	NPV
<u>IMCI guideline model</u>								
non-Case	136/3,472	Ref	3.9%	0.581 (0.580; .533,.613)	24.4%	91.8%	12.9%	96.1%
Case	44/341	3.634 (2.535,5.209)	12.9%	-				
Total	180 / 3813	-		-				
<u>WHO-composite model</u>								
non-Case	54/2,882	Ref	1.9%	0.739 (0.740; .690,.790)	70.0%	77.8%	13.5%	98.1%
Case	126/931	8.197 (5.903,11.382)	13.5%	-				
Total	180 / 3813	-		-				
<u>Independent LASSO model</u>								
-2 to -1	15/1,277	Ref	1.2%	0.791 (0.789; .742,.833)	52.8%	91.6%	23.8%	97.5%
0	23/1,164	1.696 (.881,3.266)	2.0%	-				
1-2	47/972	4.275 (2.376,7.692)	4.8%	-				
3-13	95/400	26.205 (14.988,45.819)	23.8%	-				
Total	180 / 3813			-				
<u>Composite LASSO model</u>								
-1	26/1,996	Ref	1.3%	0.781 (0.780; .725,.816)	59.4%	85.4%	16.8%	97.7%
1	9/452	1.539 (.716,3.308)	2.0%	-				
2	38/727	4.179 (2.519,6.933)	5.2%	-				
3-11	107/638	15.268 (9.842,23.686)	16.8%	-				
Total	180 / 3813							

Supplemental Table 10. Performance of clinical signs for a SpO_2<93% during outpatient pediatric care in Bangladesh and Malawi (validation dataset)

Population	Clinical sign	Prevalence SpO_2<93% N (%)	C-statistic	Sensitivity	Specificity	LR+	LR-
Overall	WHO danger signs	57 (50.9)	0.564	14	98	8.892	0.871
	Severe acute malnutrition, WAZ<-3	29 (11.7)	0.504	7	94	1.136	0.991
	Stridor	19 (45.2)	0.521	5	99	7.129	0.958
	Unable to feed	39 (61.9)	0.546	10	99	13.97	0.908
	Lethargy or unconscious	8 (47.1)	0.509	2	100	7.715	0.982
	Convulsions	8 (50)	0.509	2	100	8.616	0.982
	Severe respiratory distress with severe fast breathing	203 (28.1)	0.679	51	85	3.356	0.578
	Grunting	62 (49.2)	0.569	16	98	8.389	0.859
	Nasal flaring	137 (37.6)	0.64	35	93	5.202	0.701
	Head nodding	69 (35.2)	0.569	18	96	4.704	0.857
	Fever, temp>=38c	168 (15.8)	0.592	45	73	1.689	0.749
	Severe fast breathing: ≥70 3-11mo; ≥60 12-35mo	69 (22.5)	0.553	18	93	2.539	0.885
	Central cyanosis (lips/tongue)	25 (80.6)	0.531	6	100	36.235	0.938
	Chest indrawing	244 (20.5)	0.668	61	72	2.212	0.534
	Wheezing	65 (45.5)	0.57	16	98	7.15	0.856
3-11 months	WHO danger signs	32 (53.3)	0.567	15	98	8.88	0.864
	Severe acute malnutrition, WAZ<-3	15 (18.8)	0.516	7	96	1.793	0.968
	Stridor	8 (50)	0.517	4	100	7.838	0.967
	Unable to feed	25 (59.5)	0.554	12	99	11.419	0.892
	Lethargy or unconscious	4 (57.1)	0.509	2	100	10.596	0.983
	Convulsions	4 (50)	0.508	2	100	7.797	0.984
	Severe respiratory distress with severe fast breathing	112 (32)	0.691	53	86	3.656	0.554
	Grunting	30 (44.8)	0.56	14	98	6.356	0.877
	Nasal flaring	72 (36.9)	0.633	34	93	4.564	0.713
	Head nodding	43 (37.1)	0.58	20	96	4.609	0.833

	Fever, temp>=38c	82 (17.6)	0.588	41	76	1.747	0.769
	Severe fast breathing: $\geq$70 3-11mo; $\geq$60 12-35mo	22 (26.5)	0.535	11	96	2.88	0.928
	Central cyanosis (lips/tongue)	14 (82.4)	0.532	7	100	36.667	0.935
	Chest indrawing	140 (21.4)	0.673	66	69	2.116	0.497
	Wheezing	26 (42.6)	0.55	12	98	5.772	0.897
12-23 months	WHO danger signs	22 (57.9)	0.575	16	99	11.784	0.849
	Severe acute malnutrition, WAZ<-3	10 (9.4)	0.496	7	92	0.893	1.01
	Stridor	10 (52.6)	0.533	7	99	9.49	0.933
	Unable to feed	12 (66.7)	0.542	9	99	17.126	0.916
	Lethargy or unconscious	4 (66.7)	0.514	3	100	17.126	0.972
	Convulsions	3 (50)	0.51	2	100	8.563	0.98
	Severe respiratory distress with severe fast breathing	67 (26.6)	0.668	50	84	3.104	0.6
	Grunting	25 (58.1)	0.586	19	98	12.082	0.825
	Nasal flaring	45 (39.1)	0.638	34	94	5.551	0.707
	Head nodding	21 (36.2)	0.562	16	97	4.901	0.871
	Fever, temp>=38c	65 (16.2)	0.61	52	70	1.747	0.687
	Severe fast breathing: $\geq$70 3-11mo; $\geq$60 12-35mo	36 (22.9)	0.582	27	90	2.564	0.817
	Central cyanosis (lips/tongue)	8 (72.7)	0.529	6	100	23.178	0.942
	Chest indrawing	75 (20.9)	0.657	56	75	2.28	0.584
	Wheezing	29 (51.8)	0.596	21	98	9.205	0.804
24-35 months	WHO danger signs	3 (21.4)	0.521	6	98	3.289	0.957
	Severe acute malnutrition, WAZ<-3	4 (6.5)	0.492	8	90	0.832	1.018
	Stridor	1 (14.3)	0.505	2	99	2.044	0.989
	Unable to feed	2 (66.7)	0.52	4	100	24.612	0.961
	Lethargy or unconscious	0 (0)	0.497	0	99	0	1.007
	Convulsions	1 (50)	0.509	2	100	12.306	0.981
	Severe respiratory distress with severe fast breathing	24 (20)	0.66	48	84	3.015	0.618
	Grunting	7 (43.8)	0.563	14	99	9.349	0.873
	Nasal flaring	20 (37)	0.672	40	94	7.094	0.636
	Head nodding	5 (22.7)	0.537	10	97	3.619	0.924
	Fever, temp>=38c	21 (10.7)	0.57	44	70	1.475	0.8
	Severe fast breathing: $\geq$70 3-11mo; $\geq$60 12-35mo	11 (16.7)	0.564	22	91	2.408	0.858

Central cyanosis (lips/tongue)	3 (100)	0.531	6	100		0.939
Chest indrawing	29 (16.1)	0.665	58	75	2.316	0.56
Wheezing	10 (38.5)	0.587	20	97	7.538	0.822

Supplemental Table 11. Performance of clinical signs for a SpO_2<90% during outpatient pediatric care in Bangladesh and Malawi (validation dataset)

Population	Clinical sign	Hypoxemia prevalence (SpO_2<90%) N (%)	C-statistic	Sensitivity	Specificity	LR+	LR-
Overall	WHO danger signs	46 (41.1)	0.622	26	98	14.403	0.752
	Severe acute malnutrition, WAZ<-3	9 (3.6)	0.493	5	93	0.778	1.016
	Stridor	15 (35.7)	0.54	9	99	11.699	0.92
	Unable to feed	34 (54)	0.593	19	99	24.353	0.812
	Lethargy or unconscious	7 (41.2)	0.519	4	100	14.692	0.962
	Convulsions	6 (37.5)	0.516	3	100	12.531	0.968
	Severe respiratory distress with severe fast breathing	111 (15.4)	0.731	63	83	3.754	0.444
	Grunting	38 (30.2)	0.597	22	98	9.014	0.801
	Nasal flaring	78 (21.4)	0.684	45	92	5.663	0.602
	Head nodding	37 (18.9)	0.585	21	96	4.885	0.822
	Fever, temp>=38c	83 (7.8)	0.62	52	73	1.877	0.668
	Severe fast breathing: $\geq$70 3-11mo; $\geq$60 12-35mo	47 (15.4)	0.603	28	93	3.876	0.779
	Central cyanosis (lips/tongue)	23 (74.2)	0.566	13	100	60.659	0.868
	Chest indrawing	125 (10.5)	0.708	71	71	2.418	0.41
	Wheezing	20 (14)	0.54	11	97	3.36	0.917
3-11 months	WHO danger signs	27 (45)	0.634	29	98	15.441	0.726
	Severe acute malnutrition, WAZ<-3	4 (5)	0.5	4	96	0.993	1
	Stridor	7 (43.8)	0.536	8	99	15.085	0.928
	Unable to feed	22 (52.4)	0.611	23	99	20.748	0.775
	Lethargy or unconscious	4 (57.1)	0.521	4	100	25.934	0.958
	Convulsions	2 (25)	0.509	2	100	6.351	0.982
	Severe respiratory distress with severe fast breathing	61 (17.4)	0.743	65	84	3.983	0.419
	Grunting	21 (31.3)	0.6	23	97	8.698	0.795
	Nasal flaring	43 (22.1)	0.688	46	91	5.39	0.588
	Head nodding	23 (19.8)	0.599	25	95	4.758	0.792
	Fever, temp>=38c	36 (7.7)	0.591	43	75	1.737	0.759
	Severe fast breathing: $\geq$70 3-11mo; $\geq$60 12-35mo	14 (16.9)	0.559	16	96	4.037	0.877

	Central cyanosis (lips/tongue)	14 (82.4)	0.575	15	100	89.681	0.849
	Chest indrawing	68 (10.4)	0.697	72	67	2.19	0.413
	Wheezing	6 (9.8)	0.516	6	97	2.059	0.966
12-23 months	WHO danger signs	16 (42.1)	0.618	25	98	14.188	0.76
	Severe acute malnutrition, WAZ<-3	5 (4.7)	0.499	8	92	0.966	1.003
	Stridor	7 (36.8)	0.551	11	99	11.343	0.898
	Unable to feed	10 (55.6)	0.576	16	99	24.365	0.847
	Lethargy or unconscious	3 (50)	0.523	5	100	19.492	0.955
	Convulsions	3 (50)	0.523	5	100	19.492	0.955
	Severe respiratory distress with severe fast breathing	37 (14.7)	0.706	59	83	3.357	0.5
	Grunting	14 (32.6)	0.601	23	98	9.562	0.793
	Nasal flaring	23 (20)	0.645	37	93	4.873	0.686
	Head nodding	12 (20.7)	0.577	19	96	5.085	0.841
	Fever, temp>=38c	34 (8.5)	0.636	58	70	1.892	0.609
	Severe fast breathing: ≥70 3-11mo; ≥60 12-35mo	26 (16.6)	0.656	42	89	3.928	0.65
	Central cyanosis (lips/tongue)	8 (72.7)	0.563	13	100	52.774	0.873
	Chest indrawing	44 (12.3)	0.721	70	74	2.723	0.406
	Wheezing	12 (21.4)	0.577	19	96	5.32	0.84
24-35 months	WHO danger signs	3 (21.4)	0.57	16	98	9.1	0.857
	Severe acute malnutrition, WAZ<-3	0 (0)	0.451	0	90	0	1.108
	Stridor	1 (14.3)	0.523	6	99	5.852	0.953
	Unable to feed	2 (66.7)	0.555	11	100	70.444	0.89
	Lethargy or unconscious	0 (0)	0.497	0	99	0	1.006
	Convulsions	1 (50)	0.527	6	100	35.222	0.946
	Severe respiratory distress with severe fast breathing	13 (10.8)	0.758	68	83	4.054	0.38
	Grunting	3 (18.8)	0.569	16	98	7.676	0.86
	Nasal flaring	12 (22.2)	0.783	63	93	9.534	0.395
	Head nodding	2 (9.1)	0.54	11	97	3.522	0.918
	Fever, temp>=38c	13 (6.6)	0.714	72	70	2.447	0.394
	Severe fast breathing: ≥70 3-11mo; ≥60 12-35mo	7 (10.6)	0.638	37	91	3.953	0.696
	Central cyanosis (lips/tongue)	1 (33.3)	0.526	6	100	17.611	0.947
	Chest indrawing	13 (7.2)	0.71	68	74	2.598	0.429
	Wheezing	2 (7.7)	0.534	11	96	2.781	0.930

Supplemental Table 12. Characteristics of hypoxemic (SpO_2<93%) children indicated as wheezing (n=214)

Characteristics	Total N=214	Bangladesh N=12	Malawi N=202
Child age (months)	12 (7-17)	10.5 (7-15)	12 (7-17)
Child sex, n (%)			
Male	127 (59.3%)	11 (91.7%)	116 (57.4%)
Female	81 (37.9%)	1 (8.3%)	80 (39.6%)
Missing	6 (2.8%)	0 (0.0%)	6 (3.0%)
Weight for age z-score, n (%)			
≥-2.0	173 (80.8%)	8 (66.7%)	165 (81.7%)
-3.0≤WAZ<-2.0	22 (10.3%)	1 (8.3%)	21 (10.4%)
WAZ<-3.0	11 (5.1%)	3 (25.0%)	8 (4.0%)
Missing	8 (3.7%)	0 (0.0%)	8 (4.0%)
WHO danger signs, n (%)	55 (25.7%)	0 (0.0%)	55 (27.2%)
Stridor at rest	34 (16.3%)	0 (0.0%)	34 (17.3%)
Unable to feed	24 (11.3%)	0 (0.0%)	24 (12.0%)
Lethargy or unconscious	9 (4.2%)	0 (0.0%)	9 (4.5%)
Convulsions	9 (4.2%)	0 (0.0%)	9 (4.5%)
Severe respiratory distress	145 (68.1%)	10 (83.3%)	135 (67.2%)
Grunting	69 (33.0%)	2 (16.7%)	67 (34.0%)
Head nodding	43 (20.5%)	9 (75.0%)	34 (17.2%)
Nasal flaring	109 (51.4%)	4 (33.3%)	105 (52.5%)
Severe fast breathing[1]	32 (15.7%)	3 (25.0%)	29 (15.1%)
Central cyanosis, n (%)	10 (4.8%)	1 (8.3%)	9 (4.5%)
Temperature >38°c, n (%)	97 (48.3%)	3 (25.0%)	94 (49.7%)
Chest indrawing, n (%)	131 (61.5%)	12 (100.0%)	119 (59.2%)
Non-severe IMCI pneumonia, n (%)[2]	151 (74.4%)	9 (75.0%)	142 (74.3%)
Severe IMCI pneumonia, n (%)[3]	63 (29.4%)	3 (25.0%)	60 (29.7%)

SpO_2 indicates peripheral arterial oxyhemoglobin saturation; IQR, interquartile range; WHO, World Health Organization; IMCI, Integrated Management of Childhood Illnesses.

[1]Respiratory rate ≥ 70 breaths per minute for 3-11 month olds, ≥ 60 breaths per minute for 12-59 month olds.

[2]Cough and/or difficult breathing plus fast breathing for age (respiratory rate ≥ 50 breaths per minute for 3-11 month olds, ≥ 40 breaths per minute for 12-59 month olds) or chest wall indrawing without any WHO danger signs (stridor at rest, unable to feed, lethargy or unconscious, convulsions).

[3]Cough and/or difficult breathing plus at least one WHO danger sign (stridor at rest, unable to feed, lethargy or unconscious, convulsions), WAZ <-3, or HIV-infection with chest indrawing.
