# Supplementary figures and images for: Clinical hypoxemia score for outpatient child pneumonia care lacking pulse oximetry in Africa and South Asia
Source: Front Pediatr. 2023 Oct 4;11:1233532. doi: 10.3389/fped.2023.1233532 (PMC10582699; doi:10.3389/fped.2023.1233532)

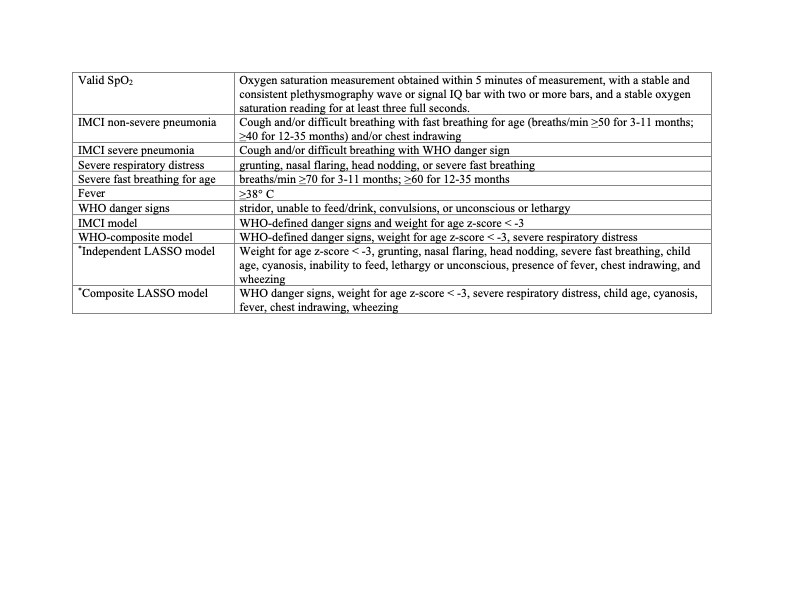

Supplement: Supplementary file 2 [file Image1.tiff]

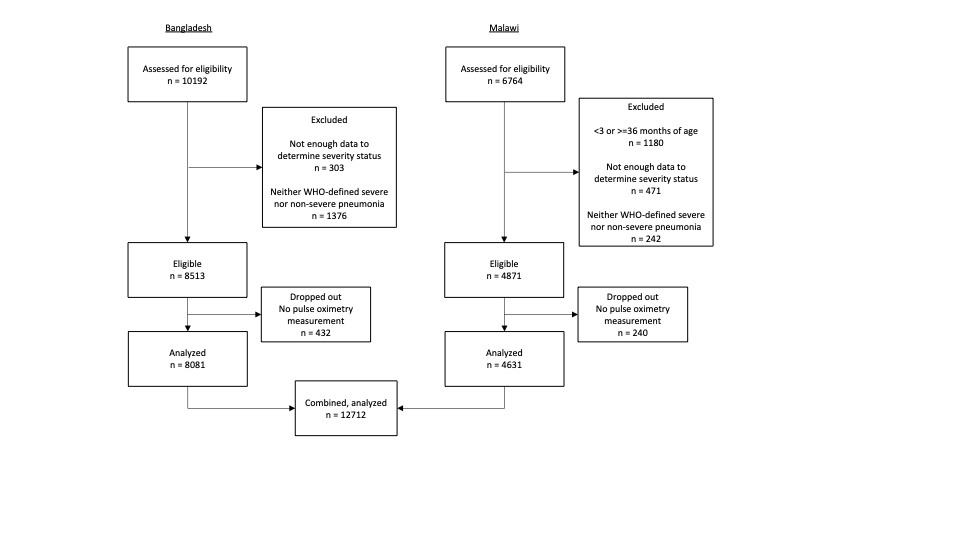

Supplement: Supplementary file 3 [file Image2.tiff]

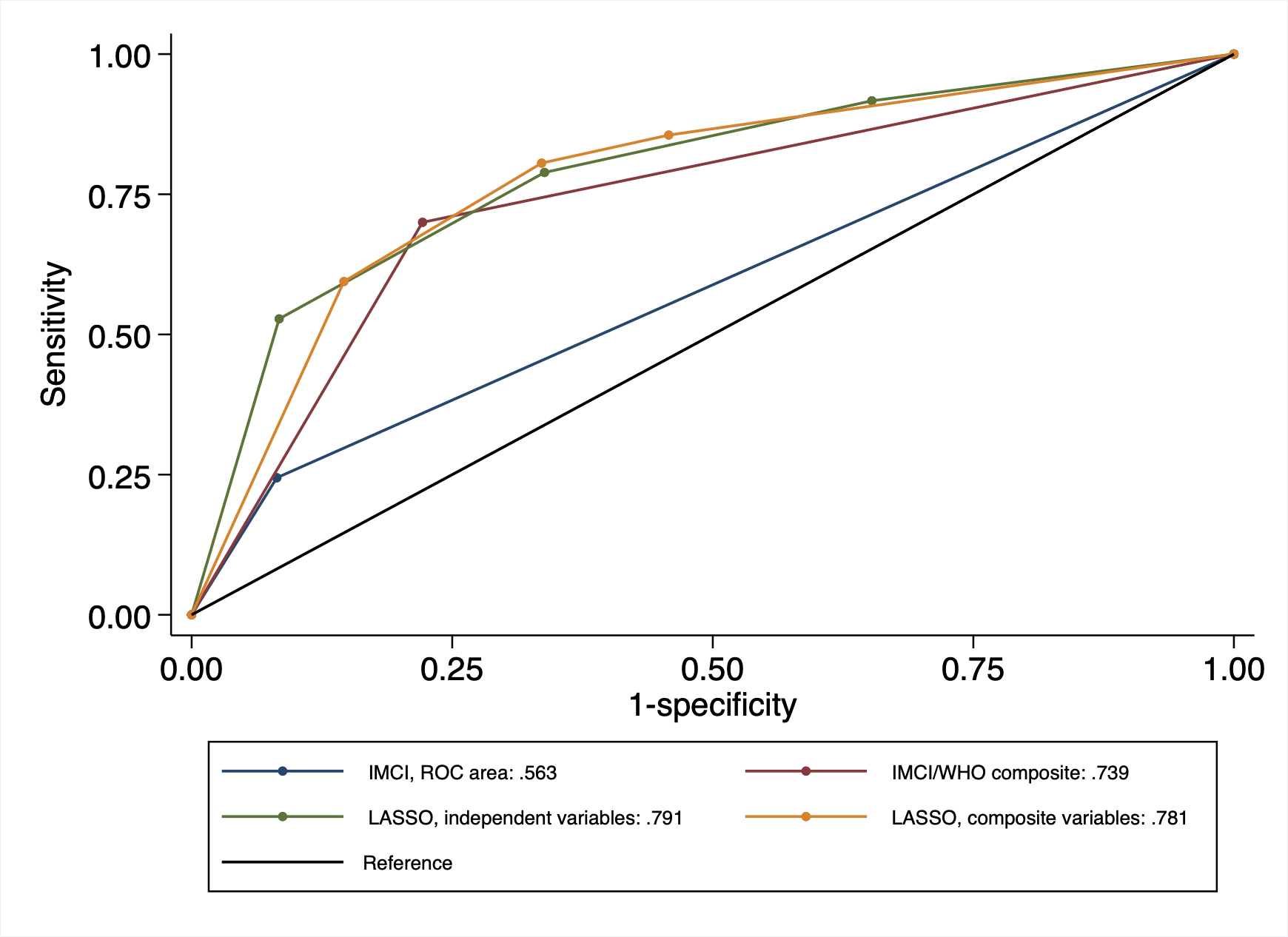

Supplement: Supplementary file 4 [file Image3.jpg]
